# Older adults in the first wave of the Corona pandemic

**DOI:** 10.1007/s10433-021-00629-3

**Published:** 2021-05-27

**Authors:** Clemens Tesch-Römer, Giovanni Lamura

**Affiliations:** 1 German Centre of Gerontology, Berlin, Germany; 2 Freie Universität Berlin, Berlin, Germany; 3 INRCA IRCCS, Centre for Socio-Economic Research on Ageing, Ancona, Italy; 4 European Centre for Social Welfare Policy and Research, Vienna, Austria

Since spring 2020, the Corona pandemic holds the world in its grip. Covid-19 has increased the death rate across all countries in the world with—in April 2021—almost 3 million deaths worldwide (WHO Covid-19 Dashboard). In the course of the pandemic, healthcare institutes and professionals came under extreme pressure. Older adults were hit hardest by the pandemic. Older people belong to those risk groups with the highest mortality risk of Covid-19, with exponential increase in the mortality risk. Beside age, the impact of the pandemic has been patterned according to social class, health status and migration status, affecting especially the lower social classes, migrants and most vulnerable people. During the first wave of the pandemic in spring and summer 2020, governmental infection control measures were aimed at physical and social distancing, shutting down shops, restaurants and theatres, where people have close contacts, as well as reducing movement of people within and across countries. By now, many countries have experienced a second wave of the pandemic and a second lockdown, and some even a third one. Shortage of masks has been overcome in many countries, but frequent testing and especially the speed of vaccinations remain critical issues, particularly for economically less privileged nations. In this situation, it is necessary to know how the living situations of older people have changed due to the pandemic itself, and the political measures meant to restrain its spread (Vieira et al. 2020).

In light of these circumstances, the European Journal of Ageing published a call for contributions on the social, behavioural and public health consequences of the Corona pandemic in later life, with the aim to inform societal stakeholders and policy makers about empirically grounded insights that may be useful to better combat the consequences of the pandemic. Many scientists from the social and behavioural sciences submitted proposals on a wide range of topics. A selection has been made, and this special section presents six empirical studies on the first wave of the pandemic, coming from different countries across the world. The dynamic of the Corona pandemic makes it almost impossible to keep track of the newest developments, especially in respect to thorough empirical studies. The six papers selected for this special section inform us about specific consequences of the first wave of the Corona pandemic in countries hit by its outbreak in the first half of the year 2020.

Perceptions of older people and ageism have been a hot topic in gerontological discourse since the onset of the pandemic. Three contributions are aimed at societal and individual images of ageing and older people. Jingjing Zhang and Xiaoting Liu have analysed the media representation of older people’s vulnerability during the COVID-19 pandemic in China. They show that media in China presented older people as passive recipients, while seeking resources from families, public institutions and governments at various levels to cope with the COVID-19 pandemic. We believe that similar observations on the role of the media can be made in other countries around the world. The study carried out by Niklas Ellerich-Groppe and colleagues investigates how the concepts of intergenerational solidarity and responsibility were used in public discourses in relation with the first COVID-19 wave. Thanks to a descriptive ethical discourse analysis of selected cases from the three areas of politics, civil society and mass media, they identify prevailing normative premises, ambiguities and biases, and their underlying assumptions about older age and intergenerational relations. In their contribution, Anna Elena Kornadt and colleagues examine older adults’ perception of ageism after the Corona outbreak and its relation to their health and well-being in Luxembourg. Their survey reveals that ageism was no rare phenomenon—being reported by one fifth of the respondents—and highlights its negative correlation with both subjective health and life satisfaction, but also the less pronounced association between ageism and subjective health for younger age groups.

Intergenerational relationships and support are in the focus of two contributions to this special issue. Bruno Arpino and colleagues report on the changes in intergenerational contacts during the COVID-19 pandemic among older people in France, Italy and Spain. In their analysis they focus on non-physical forms of contact, for instance via digital video calls. Although about 50% of older people have increased their non-physical forms of intergenerational contact during the first lockdown, a social gradient was found, where older people with lower education or less satisfying financial situation had fewer contacts. Ricardo Rodrigues and colleagues inform us about care in times of COVID-19 and on the impact of the pandemic on informal caregiving in Austria. They show that neither prevalence nor intensity of informal care changed in the course of the pandemic, but the gap in psychological well-being between carers and non-carers increased substantially.

Finally, one of the major outcomes of the pandemic is tackled in the last contribution by Honghui Pan and colleagues. They focus on the level of loneliness of older Chinese migrants in Belgium and the Netherlands during the first pandemic wave. By means of a quantitative study, they identify potential risk factors (e.g. few social contacts and feelings of threat) and protective elements (e.g. increased individual and/or non-in-person contacts and activities), and suggest that the first coronavirus outbreak may have been accompanied by increased loneliness, less social participation and financial insecurity of this specific population group.

In the meantime, preliminary evidence emerging on the impact of the successive waves of the pandemic showed that some of the initially observed findings have been further exacerbated (Paredes et al. 2021). The prolonged constraints imposed by the infection control measures, especially for most disadvantaged groups, have been affecting very different spheres of everyday life, including gender-sensitive domestic violence and home-based migrant care work, just to mention two gerontologically relevant, but socially still rather invisible phenomena (Muldoon et al. 2021). At the time being, the in-depth empirical evidence provided by the studies presented in this special section allows us to better understand the dynamics of intergenerational social cohesion in our societies, and more easily identify which efforts are needed to restore it, once the pandemic devastation will be over.

One of the crosscutting messages emerging from these studies hints to the fact that, among the areas on which attention should be prioritized—without waiting until the positive effects of the vaccination campaigns will be tangible—that of social interactions deserves a special consideration. The pandemic has deeply affected physical contacts, reducing all opportunities to enjoy, experience and face together everyday life in its multiple facets, up to a sort of “self-imposed imprisoning” by many older people into their own homes, following their legitimate stronger fear of contracting the virus (Bailey et al. 2021). This condition is further aggravated in case of frailty and of low digital skills, two additional components that contribute to make the experience of many older citizens during the pandemic as one of particular isolation (Huxhold et al. 2020). Therefore, all efforts of the coming months and years—both at political and civil society level—should aim at reactivating social interactions in all different fields, starting from the most vulnerable population groups, some of them clearly indicated by the studies presented here, such as for instance the oldest old, those with disadvantaged socio-economic position, informal carers, migrant and ethnic minority groups.

A second take-home message is that, while implementing the above indicated measures and any other necessary ones, the risk of being ageist—in one form or another—should be carefully prevented (Ayalon et al. 2020). This implies, in a first step, the acknowledgement of the presence of ageism in different areas—mass and social media, but also policy documents and civil society discourses—which, especially in the first phase of the pandemic, has sometimes depicted people in older age as passive, almost “parasitic” recipients of external help, and not always understood intergenerational solidarity as a reciprocal, symmetric responsibility between generations. As a second step, efforts should be undertaken to make sure that policies and strategies are implemented, also and particularly in this area, according to the principles of fundamental human rights and social justice, that the current pandemic has so suddenly and pervasively challenged (Larsson and Jönson 2018).

In future studies on the effect of the pandemic, we certainly need more longitudinal data which enable us to compare the situation before and after the outbreak of the pandemic. This would help to identify which aspects of the living situation are affected most by it and by the subsequent infection control measures, and which groups are more vulnerable or resilient in facing the challenges posed by the pandemic. Future research, profiting from additional, updated data considering its long-term consequences beyond the initial stage considered here, will show whether the findings highlighted by these six studies have identified just preliminary trends, that will be later partly or fully blurred by the impact of successive waves; or have rather captured long-lasting, core elements, offering a precious set of evidence-based recommendations on how to best deal with them in the—hopefully soon coming—post-pandemic times.
